# Next-Generation Sequencing Analysis of ctDNA for the Detection of Glioma and Metastatic Brain Tumors in Adults

**DOI:** 10.3389/fneur.2020.00544

**Published:** 2020-08-21

**Authors:** Jianfeng Liang, Wanni Zhao, Changyu Lu, Danni Liu, Ping Li, Xun Ye, Yuanli Zhao, Jing Zhang, Dong Yang

**Affiliations:** ^1^Department of Neurosurgery, Peking University International Hospital, Beijing, China; ^2^Department of General Surgery, Beijing Hospital, National Center of Gerontology, Beijing, China; ^3^HaploX Biotechnology, Shenzhen, China; ^4^Department of Hematology, Tongji Hospital of Tongji University, Shanghai, China; ^5^Department of Neurosurgery, Beijing Tiantan Hospital, Capital Medical University, Beijing, China; ^6^Department of Neurosurgery, China-Japan Friendship Hospital, Beijing, China; ^7^The 2nd People's Hospital of Tibet Autonomous Region, Lhasa, China

**Keywords:** ctDNA, brain tumors, NGS, MGMT, IDH1/2

## Abstract

**Background and aims:** The next-generation sequencing technologies and their related assessments of circulating tumor DNA in both glioma and metastatic brain tumors remain largely limited.

**Methods:** Based largely on a protocol approved by the institutional review board at Peking University International Hospital, the current retrospective, single-center study was conducted. Genomic DNA was extracted from blood samples or tumor tissues. With the application of NextSeq 500 instrument (Illumina), Sequencing was performed with an average coverage of 550-fold. Paired-end sequencing was employed utilized with an attempt to achieve improved sensitivity of duplicate detection and therefore to increase the detection reliability of possible fusions.

**Results:** A total of 28 patients (21 men and 7 women) with brain tumors in the present study were involved in the study. The patients enrolled were assigned into two groups, including glioma group (*n* = 21) and metastatic brain tumor group (*n* = 7). The mean age of metastatic brain tumor group (59.86 ± 8.85 y), (43.65 ± 13.05 y) reported significantly higher results in comparison to that of glioma group (45.3 ± 12.3 years) (*P* < 0.05). The mutant genes in metastatic brain tumor group included *ALK, MDM2, ATM, BRCA1, FGFR1, MDM4* and *KRAS*; however, there were no glioma-related mutant genes including *MGMT, IDH1*, IDH2, *1p/19q*, and BRAF et al. Interesteringly, only two patient (28.3%) was detected blood ctDNA in metastatic brain tumor group; In contrast, blood ctDNA was found in ten glioma patients (47.6%) including 1p/19q, *MDM2, ERBB2, IDH1, CDKN2A, CDK4, PDGFRA, CCNE1, MET*. The characterizations of *IDH* mutations in the glioma included *IDH1* mutation (p.R132H) and IDH2 mutation (p.R172K). The mutation rate of IDH in tumor tissues was 37.06 ± 8.32%, which was significantly higher than blood samples (*P* < 0.05).

**Conclusion:** The present study demonstrated that the mutant genes among glioma and metastatic brain tumors are shown to be different. Moreover, the ctDNAs in the metastatic brain tumors included *ALK* and *MDM2*, and glioma-related ctDNAs included 1p/19q and *MDM2* followed by frequencies of *ERBB2, IDH1, CDKN2A, CDK4, PDGFRA, CCNE1, MET*. These ctDNAs might be biomarkers and therapeutic responders in brain tumor.

## Background

Brain tumors are a highly heterogeneous disease with significant morbidity and mortality, which contains a collection of neoplasms arising largely from within the brain (glioma). On the other hand, brain tumors can also occur because of systemic tumors that have metastasized to the brain (metastatic brain tumors) (1). As for adults, primary brain tumors are predicted to represent 1.4% of all new cancer diagnoses and account for 2.6% of all cancer deaths (2). The overall incidence of glioma throughout the globe is estimated to be 6.4 per 100,000 persons annually, and the disease has been reported with an overall 5-year survival rate of 33.4%.

In addition, age between 55 and 64 years is considered as peak prevalence, and glioma is the most common primary brain tumor in adults (3). Metastatic brain tumors are estimated to occur as much as 10 times more frequently in comparison to glioma, which is ~53.7 per 100,000 persons (4). Either glioma or metastatic brain tumors are associated with poor prognosis (median overall survival of only 4–15 months), progressive neurological deterioration, and reduced quality of life (5, 6). Therefore, the early diagnosis, accurate differentiation, and dynamic monitoring progression of primary and metastatic brain tumors are of great importance. However, traditional methods, such as clinical examination, magnetic resonance imaging, and histopathological biopsy, are often limited to meet the requirement for clinical practice (7).

Non-invasive or minimal invasive technology to detect circulating tumor DNA (ctDNA) derived from blood (liquid biopsy) has several advantages. First, the technology is able to reduce invasive damages and avoid spatial heterogeneity and difficulties of harvesting brain tumor tissues. Second, it is more feasible and accessible, allowing for repeat blood sampling and providing dynamic insight of brain tumors progression, which becomes a promising and convincing tool to analyze the genomic characterization of brain tumors (8, 9). Recently, according to the revised fourth edition of the World Health Organization (WHO) classification of central nervous system (CNS) tumors, the integration of histology and genetic analysis for the diagnosis of specific neoplastic entities are recommended, such as isocitrate dehydrogenase 1 and 2 (*IDH1/2*) mutations, 1p/19q chromosomal codeletion, point mutations in tumor protein 53 (*TP53*), and O6-methylguanine methyltransferase (*MGMT*) promoter methylation for adults diffuse glioma (10). Other genetic alterations are meaningful for the molecular characterization of different types of brain tumors, including mutation in the promoter of telomerase reverse transcriptase (*TERT*) for oligodendroglioma, the v-RAF murine sarcoma viral oncogene homolog B1 (*BRAF*) *V600E* mutation for non-diffuse glioma, and v-rel avian reticuloendotheliosis viral oncogene homolog A (*RELA*) fusion for supratentorial ependymomas (11, 12). Recently, the next-generation sequencing (NGS) technologies have drawn increasing attention as a result of several advantages, such as globally interrogating the genetic composition of biological samples, significantly reduced sequencing cost, improved accuracy of detection, and real-time monitoring progression of tumors, with high sensitivity for detecting extremely low levels of mutation frequency; therefore, the technology allows early screening and diagnosis of brain tumors (13). However, limited reports exist considering the NGS-related assessments in both glioma and metastatic brain tumors.

In the present study, the genetic characterization of both glioma and metastatic brain tumors was comprehensively analyzed by using tumor tissue or blood samples based on the NGS technology, including mutant gene, microsatellite instability (MSI), mismatch repair, tumor mutational burden (TMB), and PD-L1 expression. Our research was conducted to help provide insight for the genetic alterations in both primary and metastatic brain tumors.

## Methods

### Participants

Based on a protocol approved by the institutional review board at Peking University International Hospital, the current retrospective, single-center study was performed according to the Good Clinical Practice guidelines, as well as the principles of the Declaration of Helsinki. The medical records of adult patients harboring brain tumors were reviewed. The patients enrolled undergoing the whole treatments in this hospital from August 2018 to June 2019 (*N* = 213). In order to be included in the current study, the following inclusion criteria should be met: (1) patients with age ranging from 18 to 75 years old; (2) patients were pathologically confirmed with primary or metastatic brain tumors, and with 5-year cancer-free history (excluding melanoma); (3) patients with normal functions of multiple vital organs (including heart, liver, lung, kidney, and bone marrow) without severe or vital illness; (4) patients scored from 0 to 1 based on the Eastern Cooperative Oncology Group (ECOG); (5) patients were in agreement with complete ctDNA tests for tissue or blood samples; (6) informed consent form was signed voluntarily by participants. The exclusion criteria included history of other malignant tumors or CNS benign tumors within 5 years (excluding melanoma), participating in other clinical trials within 3 months, organ transplant or blood transfusion recipients within 3 months, pregnant or lactating women, hepatitis B virus/hepatitis C virus/human immunodeficiency virus positive, autoimmune diseases, severe or vital illness, ECOG scoring from 2 to 5, incomplete clinical evaluations, incomplete ctDNA tests, unsigned informed consent form, and other unsuitable circumstances.

### Sampling and Sequencing

Genomic DNA was extracted from blood samples or tumor tissues. As for blood sampling, at least 10 mL of peripheral blood (anticoagulated with EDTA) was drawn from participants and separated through centrifugation (1,600 × *g*, 10 min) at room temperature. Circulating tumor DNA of blood samples was extracted with the use of QIAamp Circulating Nucleic Acid Kit (Qiagen, Hilden, Germany). As for tumor tissues, ctDNA from 10-μm formalin-fixed paraffin-embedded (FFPE) tissue was extracted through the use of QIAamp DNA FFPE Tissue Kit (Qiagen) following manufacturer's instructions. After DNA quantification, take more than 20 ng of DNA from the instructions of the kit for DNA library construction (Kapa HTP library preparation kit). The steps include ctDNA large fragment separation, small fragment recovery, DNA end repair and a-connector connection, adding special connector of Illumina sequencing kit (California, USA) at both ends of DNA, magnetic bead screening according to the required DNA fragment size, polymerase chain reaction (PCR) amplification library for probe hybridization capture, and sequencing experiment. In the panel, the target region is designed according to the reference genome sequence of Hg 19 to detect point mutation, insertion, fusion, and deletion. With the application of a NextSeq 500 instrument (Illumina), sequencing was performed with an average coverage of 550-fold. When choosing the respective adapters, the sample in the panel could be generally detected on other sequencing devices. Flow cells were selected on the basis of desired read length (150 bp), number of samples, and required target coverage for the Illumina reagent selection algorithm. The sequencing data are first processed by base calling to extract base information, and then data quality control is carried out, including removing low-quality data, tailoring data, removing poly X and other error information; data comparison, deduplication, and error correction are processed by RWA, PICARD algorithms; GATK and VarScan2 are used for variation information, genotype information, SNP, indel, et al. (14–16) are obtained. Finally, annotate the variation information. The specific methods are as follows: RAW sequencing reads were preprocessed by fastp v0.18.0 and then aligned to the reference genome (hg19/GRch37) using BWA-MEM v0.7.15 with default settings. Gencore v0.12.0 was used to remove duplicated reads. Pileup files for properly paired reads with mapping quality ≥60 were generated using Samtools v0.1.19. Somatic variants were called by VarScan2 v2.3.8 and GATK 4.0. The called somatic variants were filtered with following criteria: read depth >20 ×; variant allele frequency (VAF) ≥2% for tumor tissue DNA and ≥0.05% for cfDNA from blood samples; somatic *P* ≤ 0.01; strand filter ≥1. Allele frequencies were calculated with all bases of quality >Q30. CNVkit v0.9.3 was applied for copy number variation detection, and GeneFuse v0.6.1 was used to detect actionable gene fusions. Paired-end sequencing was employed and applied in order to improve the sensitivity of duplicate detection as well as increase the detection reliability of possible fusions (17).

### Statistics Analysis

The present study applied Statistical Product and Service Solutions software (SPSS 15.0, Inc., Chicago, IL, USA) for statistical analysis. The aggregated results were expressed as mean ± standard deviation (SD). We also utilized one-way analysis of variance (ANOVA) and two-way ANOVA and Student *t*-test for continuous data, and χ^2^ test was used for categorical data. In addition, Kruskal–Wallis test and Wilcoxon two-sample tests were used for non-normal distribution samples. *P* < 0.05 represented significant statistical difference.

## Results

### Demographic Characteristics

The current retrospective study involved a total number of 28 patients (21 men and 7 women) harboring brain tumors. The patients enrolled in the present study were divided into two groups including primary brain tumor group (*n* = 21) and metastatic brain tumor group (*n* = 7). The average age of all included 28 patients was 47.5 ± 13.8 years (range, 22–75 years). The mean age of patients in metastatic brain tumor group (61.2 ± 9.4 years) was calculated to be significantly higher when comparing that in primary brain tumor group (Age: glioma 43.65 ± 13.05, Metastatic brain tumor: 59.86 ± 8.85) (*P* < 0.05). As laid out in Table 1, the pathological type of all glioma was diffuse glioma, and the pathological types of metastatic brain tumors included lung adenocarcinoma, lung squamous carcinoma, renal cell carcinoma, intestinal adenocarcinoma, and endometrial cancer.

**Table 1:** Demographic characteristics of patients with brain tumors.

**Variable**	**All**	**Glioma tumor**	**Metastatic brain tumor**
Number, *n* (%)	28	21(75.0%)	7 (25.0%)
Age (y)	47.5 ± 13.8	43.65 ± 13.05	59.86 ± 8.85^a, b^
Gender			
Male, *n* (%)	21 (75.0 %)	16 (76.2%)	5 (71.4%)
Female, *n* (%)	7 (25.0%)	5 (23.8%)	2 (28.6%)
Pathological type, *n* (%)		Diffuse glioma, 22 (100%)	Lung adenocarcinoma, 3 (42.9%) Renal cell carcinoma, 1 (14.3%) Endometrial cancer, 1 (14.3%) Intestinal adenocarcinoma, 1 (14.3%) Lymphoma, 1(14.3%)

*Data presented as mean ± SD or n (%) and P < 0.05 was considered statistically significant*.

a *P < 0.05 vs. All*;

b *P < 0.05 vs. Primary brain tumor*.

### Data Processing Results

The results of data processing are shown in Tables 2, 3. The raw data and mapped data of cfDNA in patients' peripheral blood are shown in Table 2. All patients had more than 99.90% mapped rate and 92.00% unique mapped rate, among which patient 11 had the lowest unique mapped rate (92.40%). Patient gDNA data information is shown in Table 3. All patients had mapped rate >99.8% and unique mapped rate >98.00%.

**Table 2:** Patient cfDNA raw data and mapped data.

**Sample/cfDNA**	**Raw data**	**Clean data**	**Mapped data**			
			**Mapped reads**	**Mapped rate (%)**	**Unique mapped**	**Unique mapped rate (%)**
1	63635109	62867246	62776621	99.86	62561967	99.51
2	82706726	81569670	81522163	99.94	81442332	99.84
3	54898315	54309126	54272482	99.93	54154609	99.72
4	93141659	92242771	92196539	99.95	92045800	99.79
5	63580115	62986487	62967226	99.97	62889967	99.85
6	53309568	52834976	52820040	99.97	52767748	99.87
7	116543183	114868302	114760711	99.91	106935843	93.09
8	66168658	65159095	65082262	99.88	64932280	99.65
9	28897366	28456160	28424382	99.89	28029058	98.50
10	25802218	25289301	25238625	99.80	24949636	98.66
11	115953874	114392151	114298549	99.92	105693584	92.40
14	61003326	60465008	60446509	99.97	60377546	99.86
16	49393646	48364871	48335596	99.94	48253531	99.77
17	109574934	108354095	108284996	99.94	108180033	99.84
18	59292893	58632937	58589463	99.93	57963273	98.86
19	41871323	41284423	41268391	99.96	40798788	98.82
20	137472397	135922473	135833229	99.93	135324076	99.56
21	146248056	143746431	143605635	99.90	143424774	99.78
22	67027604	66500690	66436931	99.90	66348527	99.77
23	67628547	66881607	66857604	99.96	66774953	99.84
24	62477626	61435056	61388961	99.92	61300629	99.78
25	94187189	93238733	93186439	99.94	93078384	99.83
26	91145779	89820387	89754604	99.93	89493803	99.64
28	76629466	76077872	76011800	99.91	75816732	99.66

**Table 3:** Patient gDNA raw data and mapped data.

**Sample/gDNA**	**Raw data**	**Clean data**	**Mapped data**			
			**Mapped reads**	**Mapped rate (%)**	**Unique mapped**	**Unique mapped rate (%)**
1	66531967	65792013	65749464	99.94	65632495	99.76
2	39757181	39389673	39377443	99.97	39107803	99.28
3	35659785	34971757	34916499	99.84	34292628	98.06
4	30846537	29820218	29819198	100.00	29604650	99.28
5	29785822	29452871	29442446	99.96	29198601	99.14
6	81906016	81165609	81139259	99.97	80985694	99.78
7	50105179	49626011	49599260	99.95	49290266	99.32
8	70699970	69740194	69645649	99.86	69549091	99.73
9	43816628	43234047	43186976	99.89	42671838	98.70
10	41371341	39679044	39606057	99.82	38928846	98.11
11	111206589	109842532	109715630	99.88	109549959	99.73
12	48371223	47853335	47818179	99.93	47326009	98.90
13	45418688	42619589	42604633	99.96	42291776	99.23
14	34771950	34388704	34373003	99.95	33956643	98.74
15	114586944	110555929	110492789	99.94	109482466	99.03
16	19845365	19607222	19596907	99.95	19337713	98.63
17	47985546	47471783	47422382	99.90	45783988	96.4
18	60162428	59435412	59385430	99.92	58713449	98.79
19	42419153	41979994	41967147	99.97	41567125	99.02
20	46667933	46194675	46135923	99.87	45027230	97.47
21	80715730	79762969	79688969	99.91	79535799	99.72
22	33602295	33297637	33284888	99.96	33051096	99.26
23	36249446	35951141	35940380	99.97	35639912	99.13
24	59931926	59306786	59276075	99.95	58739233	99.04
25	100346154	99222412	99162043	99.94	98564904	99.34
26	67050678	66345831	66292803	99.92	65789276	99.16
27	28267255	27957878	27944746	99.95	27656077	98.92
28	35401850	35051830	35039088	99.96	34714611	99.04

### Genetic Alterations

**Figure 1:**
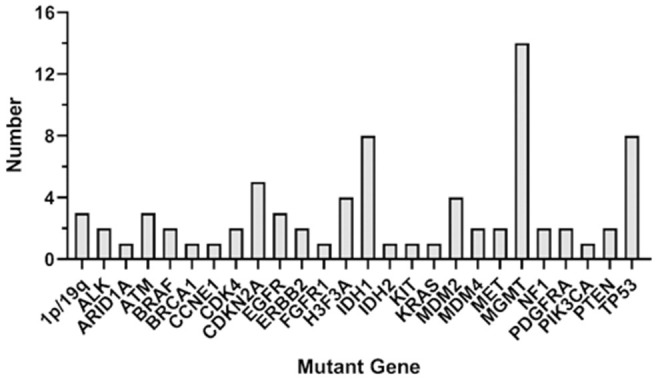
Genetic alterations in the whole participants. The figure shows the overall gene mutation statistics of 28 patients, among which MGMT has the greatest mutation probability, with a total of 14 patients; IDH1 and TP53 have the second mutation in eight patients each; CDK4 gene mutation in five patients; H3F3A and MDM2 have mutations in four patients each; 1p/19q, ALM, EGFR have mutations in three patients each, and the number of mutations in other genes is small.

As seen in Figure 1, the most frequent genetic alterations identified were *MGMT* (46.7%), followed by *IDH1* (26.7%), *TP53* (26.7%), *CDKN2A* (16.7%), *H3F3A* (13.3%), *MDM2* (13.3%), *1p/19q* (10%), *ATM* (10%), *EGFR* (10%), *ALK* (6.7%), *BRAF* (6.7%), *CDK4* (6.7%), *ERBB2* (6.7%), *MDM4* (6.7%), *MET* (6.7%), *NF1* (6.7%), *PDGFRA* (6.7%), *PTEN* (6.7%), *ARID1A* (3.3%), *BRCA1* (3.3%), *CCNE1* (3.3%), *FGFR1* (3.3%), *IDH2* (3.3%), *KIT* (3.3%), *KRAS* (3.3%), and *PIK3CA* (3.3%). Different somatic mutations occur in all genes, including amplification and fusion, chromosomal structural variation, insertion and deletion, and point mutation; among them, germline mutations occur in genes *ATM, BRCA1, IDH1, PTEN, EGFR, IDH2*, and *TP53*; ctDNA mutation rate is lower than tissue, among which *TP53, ATM, BRAF*, and *PTEN* do not occur in ctDNA.

The genetic alterations in metastatic brain tumors are shown in Table 4. The mutant genes in this group included *ALK, MDM2, ATM, BRCA1, FGFR1*, and *KRAS*. Among them, ALK mutation is the fusion of EML4-exon6 and ALK-exon 20 genes, MDM2 and FGFR1 mutation is copy number variation; ATM and BRCA1 mutations are germline heterozygous variants. The results of peripheral blood and tissue were basically similar. MDM2 did not detect variation in tissue, but the copy number in peripheral blood was 4. FGFR1 in the same patient did not detect variation in peripheral blood, but the copy number in tissue was 3.8. However, there were no glioma-related mutant genes. Remarkably, the MSI type of endometrial cancer metastatic brain tumor is MSI-H; the other MSI type of metastatic brain tumor is MSS.

**Table 4:** Genetic alternations in metastatic brain tumors.

**Pathologic types**	**Sample type**	**Mutant genes**	**Variation**	**Variation rate of tissue**	**Variation rate of peripheral blood**	**MSI**	**TMB**	**PD-L1**
Lung adenocarcinoma	Fresh tissue/peripheral blood	*ALK*,	*EML4-exon6-ALK-exon20* fusion	33.24%	49.82%	MSS	5.22	5%
		*MDM2*	Amplification		4 copies		0.87	
Lung adenocarcinoma	Fresh tissue/peripheral blood	*ALK*	*EML4-exon6-ALK-exon20* fusion	13.07%	0	MSS		–
Renal cell carcinoma	Fresh tissue/peripheral blood	*ATM*	Heterozygous (germline) c.5919-2A>G			MSS	0	–
Intestinal adenocarcinoma	Fresh tissue/peripheral blood	*KRAS*	Amplification	7.20%		MSS	2.61	1%
Lung adenocarcinoma	Fresh tissue/peripheral blood	*FGFR1*	Amplification	3.8 copies		MSS	8.7	–
Lymphoma	Fresh tissue/peripheral blood	*MDM4*	Amplification	3.8 copies	2copies	MSS	3.82	1%
Endometrial cancer	Fresh tissue/peripheral blood	*BRAC1*	Hheterozygous (germline) p.E1304fs			MSI-H	3.48	10%

**Figure 2:**
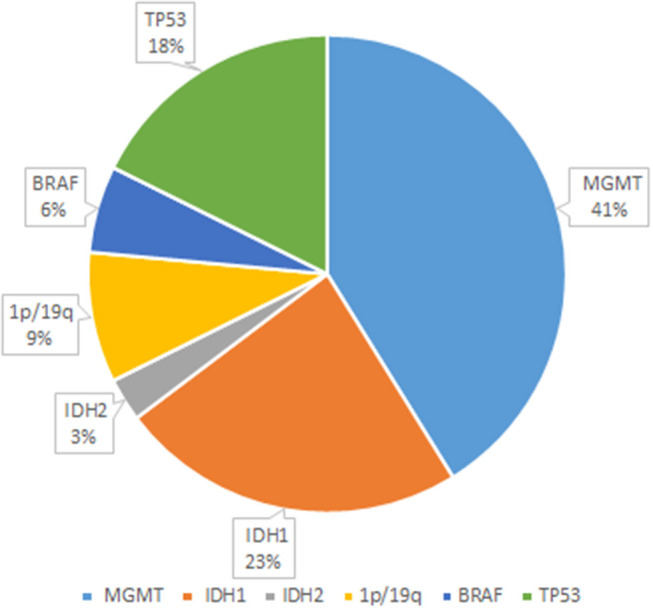
Glioma-related mutant genes. The figure shows several genes with higher probability of mutation and their respective probability of occurrence in 22 glioma patients. Among them, the genes prone to mutation were MGMT, IDH1, IDH2, 1p/19q, BRAF, and TP53, and their mutation changes were 41, 23, 3, 9, 6, and 18%, respectively.

As seen in Figure 2, glioma-related mutant genes included *MGMT* (*n* = 14), *IDH1* (*n* = 8), *IDH2* (*n* = 1), *1p/19q* (n = 3), *BRAF* (*n* = 2), and *TP53*(*n* = 6) in the glioma group. Among them, *MGMT* methylation, *IDH* mutation, *1p/19q* deletion, *BRAF* mutation, *TP53* mutation or splicing mutation, and all patients with *IDH* mutation showed *MGMT* methylation positive. All genes have different somatic mutations; among them, the genes causing germline variation are *IDH1, PTEN, EGFR, BRAF, IDH2*, and *TP53*. In the detection of glioma gene mutation, it was found that there was a great difference between the tissue mutation rate and the peripheral blood mutation rate. In general, the tissue mutation rate was higher than the peripheral blood mutation rate (Table 5). And *TP53* gene was found to be highly variable in tissue in patients with *TP53* mutation detected. Interestingly, 47.6% of glioma patients were detected ctDNA, but only two metastatic patients were found with somatic mutations in ctDNA. Glioma-related ctDNAs included 1p/19q, *MDM2, ERBB2, IDH1, CDKN2A, CDK4, PDGFRA, CCNE1, MET*. Among ctDNA positive glioma patients, 30% of them were detected 1p/19q codeletion and *MDM2* amplification in both tissue and blood.

**Table 5:** Gene mutations in tissues and peripheral blood of patients with glioma.

**Pathologic diagnosis**	**Sample type**	**Mutant gene**	**Variation**	**Variation rate of tissue (%)**	**Variation rate of peripheral blood (%)**
Glioma	Peripheral blood/Fresh tissue	*IDH1*	p.R132H	31.3	0.79
		*MGMT*	Methylation		
Glioma	Peripheral blood/Fresh tissue	*MGMT*	Methylation		
Glioma	Peripheral blood/Fresh tissue	*IDH1*	p.R132H	33.31	0
		*MGMT*	Methylation		
Glioma	Peripheral blood/Fresh tissue	*IDH1*	p.R132H	28.43	0
		*MGMT*	Methylation		
Glioma	Peripheral blood/Fresh tissue	*MGMT*	Methylation		
		*EGFR*	Amplification	44.5 copies	
		*PTEN*	p.R130*	33.76	0
		*EGFR*	p.A289v	11.67	0
Glioma	Peripheral blood/Fresh tissue	*MGMT*	Methylation		
		*IDH1*	p.R132H	31.03	0
		*1P/19q*	Codeletion	1.25 copies	2
Glioma	Peripheral blood/Fresh tissue	*MGMT*	Methylation		
		*BRAF*	p.D594N	14.89	0
		*MDM2*	Amplification	3 copies	2 copies
Glioma	Peripheral blood/Fresh tissue	*1P/19q*	Codeletion	1.26 copies	2 copies
		*IDH2*	p.R172K	36.06	0
		*MGMT*	Methylation		
Glioma	Peripheral blood/Fresh tissue	*MGMT*	Methylation		
		*1P/19q*	Codeletion	1.18 copies	2 copies
		*IDH1*	p.R172H	37.25	0
Glioma	Peripheral blood/Fresh tissue	*MGMT*	Methylation		
		*IDH1*	p.R132H	35.82	0
		*TP53*	p.H168Q	34.06	0
		*TP53*	Splice mutation	36.18	0
		*EGFR*	Amplification	27.5 copies	
Glioma	Peripheral blood/Fresh tissue	*CDKN2A*	Defect	0.4 copies	
		*PTEN*	p.N184Kfs*6	56.31	
		*MGMT*	Methylation		
		*CDK4*	Amplification	7 copies	
		*CDKN2A*	Defect	0.4 copies	
Glioma	Peripheral blood/Fresh tissue	*MGMT*	Methylation		
Glioma	Peripheral blood/Fresh tissue	*H3F3A*	p.K28M	44.24	0
		*TP53*	c.994-1G>A	85.77	0
		*CDKN2A*	Defect	1.2 copies	2 copies
		*ERBB2*	Amplification	3.9	2.1
		*MDM2*	Amplification	3.4	2
Glioma	Peripheral blood/Fresh tissue	*IDH1*	p.R132H	55.18	
		*MGMT*	Methylation		
		*TP53*	p.R273H	86.16	
		*CDKN2A*	Defect	0.4 copies	
		*MET*	Amplification	4.2 copies	
		*PDGFRA*	Amplification	79.5 copies	
		*KIT*	Amplification	79.5 copies	
Glioma	Peripheral blood/Fresh tissue	*H3F3A*	p.K28M	44.36	0
		*CDK4*	Amplification	4 copies	2 copies
		*MDM2*	Amplification	6 copies	2 copies
		*ARID1A*	p.E1787Kfs*11		
Glioma	Peripheral blood/Fresh tissue	*TP53*	p.R248Q	44.28	0
		*TP53*	p.V157L	46.99	0
		*PDGFRA*	Amplification	13.4 copies	2 copies
		*ATM*	p.I1422Qfs*4	46.34	0
		*ATM*	c.6347 + 1G > A	9.52	0
Glioma	Peripheral blood/Fresh tissue	*BRAF*	p.V600E	3.56	0
		*CCNE1*	Amplification	4 copies	2 copies
		*ERBB2*	Amplification	3 copies	2 copies
Glioma	Paraffin section/Peripheral blood	*H3F3A*	p.K28M	42.81	
		*NF1*	p.?	39.83	
		*NF1*	p.Q2507Nfs*20	33.47	
Glioma	Peripheral blood/Fresh tissue	*TP53*	p.R249	4.25	0
Glioma	Peripheral blood /FFPE	*PIK3CA*	p.H1047L	9.59	
		*H3F3A*	p.K28M	61.83	
		*MDM4*	Amplification	3 copies	
Glioma	Peripheral blood/Fresh tissue	*IDH1*	p.R132H	45.15	0
		*MGMT*	Methylation		
		*TP53*	p.R248W	91.06	0
		*MET*	Amplification	3.2	2

The genetic alterations in glioma were laid out in Table 6. The mutant genes in this group included *MGMT, IDH1*, IDH2, *1p/19q, BRAF, TP53, CDKN2A, H3F3A, MDM2, ATM, EGFR, ALK, CDK4, ERBB2, MDM4, MET, NF1, PDGFRA, PTEN, ARID1A, BRCA1, CCNE1, FGFR1, KIT, KRAS*, and *PIK3CA*. Based on Table 7, the characterizations of *IDH* mutations in the glioma included *IDH1* mutation (p.R132H) and *IDH2* mutation (p.R172K). The mutation abundance of *IDH* in tumor tissues was 37.06 ± 8.32%, which was significantly higher in comparison to that in blood samples (*P* < 0.05).

**Table 6:** Genetic alternations in primary brain tumors.

**Number**	**Mutant genes**	**MSI**	**MMR**	**TMB (muts/Mb)**	**PD-L1 (%)**
1	*IDH1, MGMT*	MSS	pMMR	3.48	–
2	*MGMT*	MSS	pMMR	3.48	–
3	*IDH1, MGMT*	MSS	pMMR	4.35	1
4	*IDH1, MGMT*	MSS	pMMR	3.48	<1
5	*MGMT, EGFR, PTEN*	MSS	pMMR	1.74	–
6	*MGMT, IDH1, 1p/19q*	MSS	pMMR	3.48	–
7	*MGMT, BRAF, MDM2*	MSS	pMMR	2.61	30
8	*MGMT. IDH2, 1p/19q*	MSI-H	pMMR	2.61	<1
9	*MGMT, IDH1, 1p/19q*	MSS	pMMR	3.48	–
10	*MGMT, IDH1, TP53*	MSS	pMMR	2.61	<1
11	*MGMT, EGFR, PTEN, CDK4, CDKN2A*	MSS	pMMR	4.35	40
12	*MGMT*	MSS	pMMR	0	1
13	*H3F3A, TP53, CDKN2A, MDM2, ERBB2*	MSS	pMMR	2.29	–
14	*MGMT, IDH1, TP53, CDKN2A, MET, KIT, PDGFRA*	MSS	pMMR	2.29	1
15	*H3F3A, CDK4, MDM2, ARID1A*	MSS	pMMR	2.29	<1
16	*TP53, PDGFRA, ATM*	MSS	pMMR	5.34	1
17	*BRAF, CCNE1, ERBB2*	MSS	pMMR	0	15
18	*NF1, H3F3A*	MSS	pMMR	3.05	<1
19	*TP53*	MSS	pMMR	0	<1
20	*H3F3A, PIK3CA, MDM4*	MSS	pMMR	1.1	1
21	*MGMT, IDH1, TP53, MET*	MSS	pMMR	1.53	<1

**Table 7:** IDH mutations in the glioma.

**Mutant gene**	**Characterization**	**Mutation Abundances in tumor tissues (%)**	**Mutation Abundances in blood (%)**
*IDH1*	p.R132H	31.30	0.79
*IDH1*	p.R132H	33.31	0
*IDH1*	p.R132H	28.43	0
*IDH1*	p.R132H	31.03	0
*IDH1*	p.R132H	37.25	0
*IDH1*	p.R132H	35.82	0
*IDH1*	p.R132H	55.18	0
*IDH1*	p.R132H	45.15	0
*IDH2*	p.R172K	36.06	0

## Discussion

The accurate differentiation of primary and metastatic brain tumors is considered pivotal, considering that the intervention and therapy approaches for patients with these two types of tumors are remarkably different for clinical practice (18, 19). The cancers with the highest propensity in terms of metastasizing to the brain are lung (50%), followed by breast (15%) and melanoma (5–10%), accounting for ~80% of all brain metastases (20). In this study, we found that the mutant genes in the metastatic brain tumors included *ALK, MDM2, ATM, BRCA1, FGFR1*, and *KRAS*, and there were no glioma-related mutant genes (*MGMT, IDH1, IDH2, 1p/19q, BRAF*, and *TERT*). According to the aggregated result, NGS-based genetic analysis might become a promising tool to differentiate primary and metastatic brain tumors. Based on a report supported by Bettegowda et al. (21), the sensitivity of ctDNA was 87.2% for detection of clinically relevant *KRAS* gene mutations, with specificity of 99.2% in the detection of metastatic cancers. Wang et al. (22) suggested that liquid biopsy such as ctDNA could be regarded a feasible alternative approach in terms of identifying sensitizing genomic alterations, and ALK translocation could be identified in the diffuse brain metastases. However, in this study ALK, MEM2 and MDM4 were detected in ctDNA of only two brain metastatic patients. It might be due to sample size, and we plan to expand the sample size in further study. Moreover, the results showed that glioma-related mutant genes included *MGMT* (*n* = 14), *IDH1* (*n* = 8), *IDH2* (*n* = 1), *1p/19q* (*n* = 3), and *BRAF* (*n* = 2). As a DNA repair protein, MGMT is able to remove the alkylation of the O6 position of guanine which is also the most cytotoxic lesion induced by alkylating agent chemotherapy (23). Hypermethylation of the promoter of *MGMT* is considered to have predictive value to respond to the alkylating agent temozolomide among patients harboring glioblastoma (24). Piccioni et al. (25) reported that 50% of patients with glioma had ≥1 somatic alteration detected. Additionally, 61 genes were found with single-nucleotide variants, and amplifications were detected in *EGFR MET, ERBB2*, and others, indicating that plasma cfDNA genomic analysis might be used as a viable approach for clinical practice to identify genomically driven therapy options. According to the study of Schwaederle et al. (26), the most frequent alterations among diverse cancers were reported to be *TP53* (29.8%), followed, respectively, by *EGFR* (17.5%), *MET* (10.5%), *PIK3CA* (7%), and *NOTCH1* (5.8%). In addition, detectable ctDNA aberrations existed among 65% of diverse cancers (as well as 27% of glioblastomas), with the majority theoretically actionable by an approved agent. In this study, 47.6% of glioma patients were detected ctDNA including 1p/19q, *MDM2, ERBB2, IDH1, CDKN2A, CDK4, PDGFRA, CCNE1, MET*. These ctDNAs might be biomarkers and therapeutic responders in glioma and be worthy of further investigation.

Furthermore, we found that the characterizations of *IDH* mutations in the glioma included *IDH1* mutation (p.R132H) and *IDH2* mutation (p.R172K). The mutation abundance of *IDH* in tumor tissues was 37.06 ± 8.32%, which reported evidently higher results in comparison to that in blood samples (*P* < 0.05). *IDH*-mutated results were found in at least 80% of WHO grades II and III infiltrating astrocytomas and secondary GBMs, whereas all oligodendrogliomas were *IDH*-mutated and 1p/19q co-deleted (27). Similar results were observed in the study of Hartmann et al. (28) that codon 132 of the *IDH1* gene, known as the R132H variant, was reported to account for 92.7% of *IDH* mutation, followed by R132C (4.1%), R132S (1.5%), R132G (1.4%), and R132L (0.2%). Moreover, residue R172 in exon 4 of the *IDH2* gene was homologous to R132 in the *IDH1* gene, and the most common *IDH2* mutations included R172W (16%), R172M (19%), and R172K (65%). In current study, three glioma patients were oligodendrogliomas, and 1p/19q codeletion was detected in both blood and tissue of these patients. Interestingly, copy number of 1p/19q is higher in blood than tissue. Thus these findings provide new insights into verification 1p/19q codeleciton to glioma patient via a noninvasive approach. Lots of focus has been paid to the predictive value of TMB as biomarker in terms of the response to immune checkpoint blockade therapy among many clinical trials (29). High TMB was consistently selected for beneficial outcome with immune checkpoint blockade therapy, and we found that the mean TMB of glioma was 2.55 mutations per Mb. According to the study supported by Johnson et al. (30), 43 to 575 mutations per Mb existed in hypermutated gliomas characterized by TMBs.

## Conclusion

The present study demonstrated that the mutant genes among glioma and metastatic brain tumors include are different. Moreover, the ctDNAs in the metastatic brain tumors included *ALK* and *MDM2*, and glioma-related ctDNAs included 1p/19q and *MDM2* followed by frequencies of *ERBB2, IDH1, CDKN2A, CDK4, PDGFRA, CCNE1, MET*. These ctDNAs might be noninvasive biomarkers and therapeutic responders in brain tumor.

## Data Availability Statement

The datasets presented in this study can be found in online repositories. The names of the repository/repositories and accession number(s) can be found below: https://bigd.big.ac.cn/search?dbId=&q=HRA000141, with accession no: HRA000141.

## Ethics Statement

The studies involving human participants were reviewed and approved by Institutional Review Board at Peking University International Hospital. The patients/participants provided their written informed consent to participate in this study.

## Author Contributions

JL, WZ, CL, and DL carried out the experiments. DL, PL, and JZ participated in data analysis. CL, XY, and YZ participated in the clinical investigation of the patient. JL, PL, and DY drafted the manuscript. JL, JZ, and DY offered opinions for discussions and reviewed the manuscript. YZ and DY critically reviewed the overall manuscript as well as supervised the study. All authors contributed to the article and approved the submitted version.

## Conflict of Interest

The authors declare that the research was conducted in the absence of any commercial or financial relationships that could be construed as a potential conflict of interest.
